# 5-[4-(1*H*-Imidazol-1-yl)phen­yl]-2*H*-tetra­zole dihydrate

**DOI:** 10.1107/S1600536811019647

**Published:** 2011-05-28

**Authors:** Yan-Hua Zhang, Da-Min Tian

**Affiliations:** a Department of Chemistry and Chemical Engineering, Henan University of Urban Construction, Pingdingshan, Henan, People’s Republic of China

## Abstract

In the title compound, C_10_H_8_N_6_·2H_2_O, the central aromatic ring makes dihedral angles of 23.59 (15) and 16.99 (16)° with the terminal imidazole and tetra­zole rings, respectively, which are themselves almost coplanar [dihedral angle = 6.61 (18)°]. Two H atoms of the two water mol­ecules are half occupied. In the crystal packing, weak inter­molecular O—H⋯N, O—H⋯O and N—H⋯N hydrogen bonds and π–π stacking inter­actions [centroid–centroid distances of 3.73 (4) Å between benzene rings and 3.66 (3) Å between imidazole and tetra­zole rings] are observed.

## Related literature

For the biological activity of imidazole derivatives, see: Reichardt *et al.* (1992 ▶)
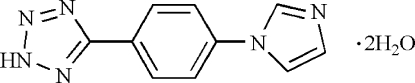


## Experimental

### 

#### Crystal data

C_10_H_8_N_6_·2H_2_O
*M* *_r_* = 248.26
Triclinic, 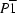

*a* = 7.4300 (7) Å
*b* = 8.2285 (9) Å
*c* = 10.2047 (11) Å
α = 97.011 (1)°
β = 90.813 (1)°
γ = 113.449 (2)°
*V* = 566.74 (10) Å^3^
*Z* = 2
Mo *K*α radiation
μ = 0.11 mm^−1^
*T* = 298 K
0.38 × 0.17 × 0.16 mm

#### Data collection

Bruker SMART 1000 CCD area-detector diffractometer
Absorption correction: multi-scan (*SADABS*; Bruker, 2004 ▶) *T*_min_ = 0.960, *T*_max_ = 0.983
2913 measured reflections
1953 independent reflections
1199 reflections with *I* > 2σ(*I*)
*R*_int_ = 0.024

#### Refinement

*R*[*F* ^2^ > 2σ(*F* ^2^)] = 0.075
*wR*(*F* ^2^) = 0.243
*S* = 1.05
1953 reflections
164 parameters
H-atom parameters constrained
Δρ_max_ = 0.40 e Å^−3^
Δρ_min_ = −0.51 e Å^−3^

### 

Data collection: *SMART* (Bruker, 2004 ▶); cell refinement: *SAINT* (Bruker, 2004 ▶); data reduction: *SAINT*; program(s) used to solve structure: *SHELXS97* (Sheldrick, 2008 ▶); program(s) used to refine structure: *SHELXL97* (Sheldrick, 2008 ▶); molecular graphics: *SHELXTL* (Sheldrick, 2008 ▶); software used to prepare material for publication: *SHELXTL*.

## Supplementary Material

Crystal structure: contains datablocks I, global. DOI: 10.1107/S1600536811019647/jh2291sup1.cif

Structure factors: contains datablocks I. DOI: 10.1107/S1600536811019647/jh2291Isup2.hkl

Supplementary material file. DOI: 10.1107/S1600536811019647/jh2291Isup3.cml

Additional supplementary materials:  crystallographic information; 3D view; checkCIF report

## Figures and Tables

**Table 1:** Hydrogen-bond geometry (Å, °)

*D*—H⋯*A*	*D*—H	H⋯*A*	*D*⋯*A*	*D*—H⋯*A*
O2—H2*D*⋯N2^i^	0.85	2.50	3.235 (7)	146
O2—H2*D*⋯N3^i^	0.85	1.98	2.823 (7)	172
O2—H2*C*⋯O1^ii^	0.85	2.15	2.994 (9)	170
O2—H2*A*⋯O1	0.85	1.92	2.629 (9)	141
O1—H1*D*⋯O2^ii^	0.85	2.55	2.994 (9)	114
O1—H1*D*⋯O2	0.85	1.78	2.629 (9)	175
O1—H1*C*⋯O1^iii^	0.85	1.96	2.806 (11)	175
O1—H1*A*⋯N1	0.85	1.99	2.790 (5)	157
N2—H2⋯N6^iv^	0.86	1.90	2.758 (5)	174

Symmetry codes: (i) 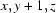; (ii) 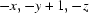; (iii) 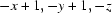; (iv) 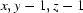.

**Fig. 1.**
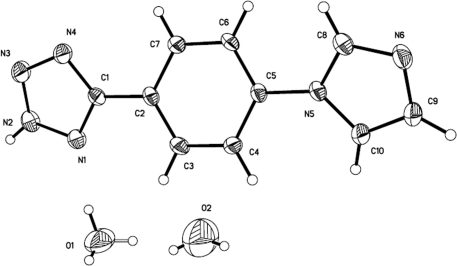
The structure of the title compound, showing the atomic numbering scheme with 30% probability displacement ellipsoids

## supplementary crystallographic information

### Comment

Imidazole derivatives possessing the biologically important imdidazole ring, have been studied in terms of their biological activities (Reichardt *et al.*, 1992). Inspired by this, we focus on the studies of imidazole derivatives Recently, we have obtain a new crystal structure of imidazole derivative (1-tetrazole-4-imidazole-benzene), which is crystallized by the slow evaporation of ethonal sovlent at room temperaure.

As shown in Fig.1, the title molecule crystallizes as a neutral, with two terminal imidazole and tetrazole rings almost coplanar with the dihedral angle of 6.61 (18) °. They makes dihedral angles of 23.59 (15) ° and 16.99 (16) ° with the central aromatic ring. It is noted that there are two types of π-π stacking interactions: one occurs between parallel benzene rings with centroid-centroid distances of 3.73 (4) Å; the other occurs between the imidazole and tetrazole rings with centroid-centroid distances of 3.66 (3) Å. Thus, A wide range of hydrogen bonds (O—H···N, O—H···O and N—H···N) and π-π stacking interactions contribute to the formation of the supramolecular network.

### Experimental

1-tetrazole-4-imidazole-benzene(0.1 g, 0.4 mmol) was dissolved in ethonal (20 ml) and the solution was left to evaporate slowly at room temperature. After a week, colourless crystals suitable for X-ray analysis were obtained.

### Refinement

Carboxyl H atoms were located in a difference map but were refined as riding on the parent O atoms with O—H = 0.82 Å and *U*_iso_(H) = 1.5 *U*_eq_(O). Carbon and nitrogen bound H atoms were placed at calculated positions and were treated as riding on the parent C or N atoms with C—H = 0.96 (methyl), 0.97 (methylene) and N—H = 0.86 Å, *U*_iso_(H) = 1.2 or 1.5 *U*_eq_(C, N). H atoms of the water molecule were located in a difference Fourier map and refined as riding with an O—H distance restraint of 0.84 (1) Å, with *U*_iso_(H) = 1.5 *U*_eq_. two hydgrogen atoms from two water molecules are half ocuppied and split into two atoms, respectively.

### Crystal data

**Table:**

C_10_H_8_N_6_·2H_2_O	*Z* = 2
*M**_r_* = 248.26	*F*(000) = 260
Triclinic, *P*1	*D*_x_ = 1.455 Mg m^−^^3^
Hall symbol: -P 1	Mo *K*α radiation, λ = 0.71073 Å
*a* = 7.4300 (7) Å	Cell parameters from 1702 reflections
*b* = 8.2285 (9) Å	θ = 2.5–25.9°
*c* = 10.2047 (11) Å	µ = 0.11 mm^−^^1^
α = 97.011 (1)°	*T* = 298 K
β = 90.813 (1)°	Block, colorless
γ = 113.449 (2)°	0.38 × 0.17 × 0.16 mm
*V* = 566.74 (10) Å^3^	

### Data collection

**Table:**

Bruker SMART 1000 CCD area-detector diffractometer	1953 independent reflections
Radiation source: fine-focus sealed tube	1199 reflections with *I* > 2σ(*I*)
graphite	*R*_int_ = 0.024
φ and ω scans	θ_max_ = 25.0°, θ_min_ = 3.2°
Absorption correction: multi-scan (*SADABS*; Bruker, 2004)	*h* = −8→8
*T*_min_ = 0.960, *T*_max_ = 0.983	*k* = −9→9
2913 measured reflections	*l* = −11→12

### Refinement

**Table:**

Refinement on *F*^2^	Secondary atom site location: difference Fourier map
Least-squares matrix: full	Hydrogen site location: inferred from neighbouring sites
*R*[*F*^2^ > 2σ(*F*^2^)] = 0.075	H-atom parameters constrained
*wR*(*F*^2^) = 0.243	*w* = 1/[σ^2^(*F*_o_^2^) + (0.1194*P*)^2^ + 0.5477*P*] where *P* = (*F*_o_^2^ + 2*F*_c_^2^)/3
*S* = 1.05	(Δ/σ)_max_ < 0.001
1953 reflections	Δρ_max_ = 0.40 e Å^−^^3^
164 parameters	Δρ_min_ = −0.51 e Å^−^^3^
0 restraints	Extinction correction: *SHELXL97* (Sheldrick, 2008), Fc^*^=kFc[1+0.001xFc^2^λ^3^/sin(2θ)]^-1/4^
Primary atom site location: structure-invariant direct methods	Extinction coefficient: 0.13 (2)

### Special details

**Table:**

Geometry. All e.s.d.'s (except the e.s.d. in the dihedral angle between two l.s. planes) are estimated using the full covariance matrix. The cell e.s.d.'s are taken into account individually in the estimation of e.s.d.'s in distances, angles and torsion angles; correlations between e.s.d.'s in cell parameters are only used when they are defined by crystal symmetry. An approximate (isotropic) treatment of cell e.s.d.'s is used for estimating e.s.d.'s involving l.s. planes.
Refinement. Refinement of *F*^2^ against ALL reflections. The weighted *R*-factor *wR* and goodness of fit *S* are based on *F*^2^, conventional *R*-factors *R* are based on *F*, with *F* set to zero for negative *F*^2^. The threshold expression of *F*^2^ > σ(*F*^2^) is used only for calculating *R*-factors(gt) *etc*. and is not relevant to the choice of reflections for refinement. *R*-factors based on *F*^2^ are statistically about twice as large as those based on *F*, and *R*- factors based on ALL data will be even larger.

### Fractional atomic coordinates and isotropic or equivalent isotropic displacement parameters (Å^2^)

**Table:**

	*x*	*y*	*z*	*U*_iso_*/*U*_eq_	Occ. (<1)
N1	0.2591 (5)	0.1994 (4)	0.1928 (3)	0.0420 (9)	
N2	0.2495 (6)	0.0363 (4)	0.1481 (3)	0.0483 (10)	
H2	0.2561	−0.0008	0.0665	0.058*	
N3	0.2288 (6)	−0.0589 (5)	0.2440 (3)	0.0499 (10)	
N4	0.2239 (6)	0.0414 (4)	0.3577 (3)	0.0457 (10)	
N5	0.2568 (4)	0.7806 (4)	0.6934 (3)	0.0329 (8)	
N6	0.2612 (5)	0.9372 (4)	0.8813 (3)	0.0447 (10)	
O1	0.2945 (8)	0.4108 (6)	−0.0073 (4)	0.1116 (18)	
H1A	0.2650	0.3229	0.0363	0.134*	
H1C	0.4182	0.4705	−0.0013	0.134*	0.50
H1D	0.2401	0.4795	0.0231	0.134*	0.50
O2	0.1048 (12)	0.6070 (9)	0.0820 (6)	0.183 (3)	
H2A	0.2078	0.5941	0.0576	0.220*	0.50
H2C	−0.0111	0.5894	0.0548	0.220*	0.50
H2D	0.1533	0.7067	0.1322	0.220*	
C1	0.2426 (5)	0.1985 (5)	0.3235 (3)	0.0320 (9)	
C2	0.2436 (5)	0.3498 (5)	0.4159 (3)	0.0304 (9)	
C3	0.3121 (6)	0.5229 (5)	0.3835 (3)	0.0377 (10)	
H3	0.3556	0.5438	0.2997	0.045*	
C4	0.3163 (6)	0.6638 (5)	0.4737 (4)	0.0367 (10)	
H4A	0.3632	0.7788	0.4508	0.044*	
C5	0.2507 (5)	0.6338 (5)	0.5985 (3)	0.0296 (9)	
C6	0.1766 (6)	0.4619 (5)	0.6316 (3)	0.0372 (10)	
H6	0.1290	0.4413	0.7144	0.045*	
C7	0.1736 (6)	0.3217 (5)	0.5418 (3)	0.0374 (10)	
H7	0.1245	0.2067	0.5648	0.045*	
C8	0.2597 (6)	0.7830 (6)	0.8248 (4)	0.0416 (11)	
H8	0.2604	0.6911	0.8692	0.050*	
C9	0.2610 (6)	1.0379 (5)	0.7849 (4)	0.0436 (11)	
H9	0.2632	1.1526	0.7977	0.052*	
C10	0.2571 (6)	0.9415 (5)	0.6680 (4)	0.0424 (11)	
H10	0.2550	0.9768	0.5850	0.051*	

### Atomic displacement parameters (Å^2^)

**Table:**

	*U*^11^	*U*^22^	*U*^33^	*U*^12^	*U*^13^	*U*^23^
N1	0.059 (2)	0.0374 (19)	0.0299 (17)	0.0204 (17)	0.0075 (15)	−0.0007 (14)
N2	0.075 (3)	0.044 (2)	0.0252 (17)	0.027 (2)	0.0086 (16)	−0.0097 (15)
N3	0.077 (3)	0.043 (2)	0.0345 (18)	0.032 (2)	0.0062 (17)	−0.0028 (16)
N4	0.070 (3)	0.0366 (19)	0.0340 (18)	0.0267 (18)	0.0090 (16)	0.0007 (15)
N5	0.0396 (19)	0.0335 (18)	0.0248 (16)	0.0156 (15)	0.0047 (13)	−0.0015 (13)
N6	0.053 (2)	0.045 (2)	0.0346 (18)	0.0218 (18)	0.0061 (15)	−0.0048 (16)
O1	0.194 (5)	0.088 (3)	0.069 (3)	0.067 (3)	0.034 (3)	0.033 (2)
O2	0.248 (9)	0.142 (6)	0.156 (6)	0.078 (6)	0.023 (6)	0.004 (5)
C1	0.035 (2)	0.036 (2)	0.0244 (18)	0.0141 (17)	0.0037 (15)	0.0017 (16)
C2	0.032 (2)	0.036 (2)	0.0239 (18)	0.0159 (17)	0.0013 (14)	−0.0017 (15)
C3	0.049 (3)	0.041 (2)	0.0230 (18)	0.0178 (19)	0.0084 (16)	0.0051 (16)
C4	0.050 (3)	0.028 (2)	0.031 (2)	0.0137 (18)	0.0091 (17)	0.0055 (16)
C5	0.033 (2)	0.031 (2)	0.0250 (18)	0.0151 (17)	0.0019 (14)	−0.0005 (15)
C6	0.050 (3)	0.039 (2)	0.0242 (18)	0.019 (2)	0.0149 (16)	0.0045 (16)
C7	0.052 (3)	0.032 (2)	0.0300 (19)	0.0187 (19)	0.0100 (17)	0.0056 (16)
C8	0.054 (3)	0.043 (2)	0.0271 (19)	0.020 (2)	0.0049 (17)	−0.0005 (17)
C9	0.057 (3)	0.036 (2)	0.040 (2)	0.023 (2)	0.0051 (19)	−0.0023 (18)
C10	0.061 (3)	0.036 (2)	0.033 (2)	0.023 (2)	0.0077 (18)	0.0022 (17)

### Geometric parameters (Å, °)

**Table:**

N1—N2	1.337 (5)	O2—H2D	0.8501
N1—C1	1.341 (5)	C1—C2	1.465 (5)
N2—N3	1.300 (5)	C2—C3	1.393 (5)
N2—H2	0.8600	C2—C7	1.403 (5)
N3—N4	1.350 (5)	C3—C4	1.379 (5)
N4—C1	1.335 (5)	C3—H3	0.9300
N5—C8	1.338 (5)	C4—C5	1.386 (5)
N5—C10	1.379 (5)	C4—H4A	0.9300
N5—C5	1.438 (5)	C5—C6	1.385 (5)
N6—C8	1.324 (5)	C6—C7	1.375 (5)
N6—C9	1.362 (5)	C6—H6	0.9300
O1—H1A	0.8500	C7—H7	0.9300
O1—H1C	0.8500	C8—H8	0.9300
O1—H1D	0.8499	C9—C10	1.343 (5)
O2—H2A	0.8500	C9—H9	0.9300
O2—H2C	0.8500	C10—H10	0.9300
			
N2—N1—C1	104.1 (3)	C4—C3—H3	119.5
N3—N2—N1	111.2 (3)	C2—C3—H3	119.5
N3—N2—H2	124.4	C3—C4—C5	119.9 (3)
N1—N2—H2	124.4	C3—C4—H4A	120.1
N2—N3—N4	108.3 (3)	C5—C4—H4A	120.1
C1—N4—N3	105.4 (3)	C6—C5—C4	120.0 (3)
C8—N5—C10	107.3 (3)	C6—C5—N5	119.9 (3)
C8—N5—C5	125.3 (3)	C4—C5—N5	120.1 (3)
C10—N5—C5	127.4 (3)	C7—C6—C5	120.0 (3)
C8—N6—C9	108.6 (3)	C7—C6—H6	120.0
H1A—O1—H1C	110.2	C5—C6—H6	120.0
H1A—O1—H1D	110.2	C6—C7—C2	120.9 (4)
H1C—O1—H1D	108.5	C6—C7—H7	119.6
H2A—O2—H2C	142.7	C2—C7—H7	119.6
H2A—O2—H2D	101.7	N6—C8—N5	109.1 (3)
H2C—O2—H2D	108.1	N6—C8—H8	125.5
N4—C1—N1	111.1 (3)	N5—C8—H8	125.5
N4—C1—C2	124.6 (3)	C10—C9—N6	107.5 (4)
N1—C1—C2	124.3 (3)	C10—C9—H9	126.2
C3—C2—C7	118.1 (3)	N6—C9—H9	126.2
C3—C2—C1	122.3 (3)	C9—C10—N5	107.5 (3)
C7—C2—C1	119.6 (3)	C9—C10—H10	126.2
C4—C3—C2	121.0 (3)	N5—C10—H10	126.2
			
C1—N1—N2—N3	−0.1 (5)	C8—N5—C5—C6	23.6 (6)
N1—N2—N3—N4	0.1 (5)	C10—N5—C5—C6	−155.1 (4)
N2—N3—N4—C1	0.0 (5)	C8—N5—C5—C4	−157.2 (4)
N3—N4—C1—N1	−0.1 (5)	C10—N5—C5—C4	24.1 (6)
N3—N4—C1—C2	179.5 (4)	C4—C5—C6—C7	1.9 (6)
N2—N1—C1—N4	0.1 (4)	N5—C5—C6—C7	−178.9 (3)
N2—N1—C1—C2	−179.5 (3)	C5—C6—C7—C2	−0.4 (6)
N4—C1—C2—C3	163.4 (4)	C3—C2—C7—C6	−1.4 (6)
N1—C1—C2—C3	−17.1 (6)	C1—C2—C7—C6	178.6 (4)
N4—C1—C2—C7	−16.6 (6)	C9—N6—C8—N5	−0.5 (5)
N1—C1—C2—C7	162.9 (4)	C10—N5—C8—N6	0.2 (5)
C7—C2—C3—C4	1.8 (6)	C5—N5—C8—N6	−178.7 (3)
C1—C2—C3—C4	−178.2 (3)	C8—N6—C9—C10	0.7 (5)
C2—C3—C4—C5	−0.4 (6)	N6—C9—C10—N5	−0.6 (5)
C3—C4—C5—C6	−1.5 (6)	C8—N5—C10—C9	0.3 (5)
C3—C4—C5—N5	179.3 (3)	C5—N5—C10—C9	179.1 (4)

### Hydrogen-bond geometry (Å, °)

**Table:**

*D*—H···*A*	*D*—H	H···*A*	*D*···*A*	*D*—H···*A*
O2—H2D···N2^i^	0.85	2.50	3.235 (7)	146
O2—H2D···N3^i^	0.85	1.98	2.823 (7)	172
O2—H2C···O1^ii^	0.85	2.15	2.994 (9)	170
O2—H2A···O1	0.85	1.92	2.629 (9)	141
O1—H1D···O2^ii^	0.85	2.55	2.994 (9)	114
O1—H1D···O2	0.85	1.78	2.629 (9)	175
O1—H1C···O1^iii^	0.85	1.96	2.806 (11)	175
O1—H1A···N1	0.85	1.99	2.790 (5)	157
N2—H2···N6^iv^	0.86	1.90	2.758 (5)	174

Symmetry codes: (i) *x*, *y*+1, *z*; (ii) −*x*, −*y*+1, −*z*; (iii) −*x*+1, −*y*+1, −*z*; (iv) *x*, *y*−1, *z*−1.

## References

[bb1] Bruker (2004). *SMART*, *SAINT* and *SADABS* Bruker AXS Inc., Madison, Wisconsin, USA.

[bb2] Reichardt, B. A., Belyavtseva, L. M. & Kulikova, O. G. (1992). *Bull. Exp. Biol. Med.* **113**, 506–508.

[bb3] Sheldrick, G. M. (2008). *Acta Cryst.* A**64**, 112–122. 10.1107/S0108767307043930 18156677

